# T-cell replete haploidentical transplantation compared to mismatched unrelated allogeneic transplantation with post-transplantation cyclophosphamide in patients with secondary acute myeloid leukemia in first complete remission: A study from the ALWP/EBMT

**DOI:** 10.1038/s41409-026-02951-9

**Published:** 2026-06-27

**Authors:** Sarah Kayser, Arnon Nagler, Allain Thibeault Ferhat, Jaime Sanz, Raynier Devillier, Yener Koc, Anna Maria Raiola, Alessandro Busca, Alexander Kulagin, Jan Vydra, Maija Itala-Remes, Stefania Bramanti, Giovanni Grillo, Zafer Gulbas, Jiri Pavlu, Montserrat Rovira, Simona Sica, Alessandro Rambaldi, Eolia Brissot, Ali Bazarbachi, Mohamad Mohty, Fabio Ciceri

**Affiliations:** 1 Department of Hematology, Oncology and Cancer Immunology, Charité-Universitätsmedizin Berlin, corporate member of Freie Universität Berlin and Humboldt-Universität zu Berlin, Berlin, Germany; 2 Division of Hematology, Sheba Medical Center, Tel Hashomer and Tel Aviv University, Tel Aviv, Israel; 3 EBMT Paris study office; Department of Haematology, Saint Antoine Hospital; INSERM UMR 938, Sorbonne University, Paris, France; 4 Hematology Department, Hospital Universitari i Politècnic La Fe, Departament de Medicina Universitat de Valencia, de Valencia, Spain; 5 Transplantation and cellular immunotherapy program, Department of Hematology, Institut Paoli Calmettes, Management Sport Cancer Lab, Aix Marseille Univ, Marseille, France; 6 Medicana International Hospital Istanbul, Istanbul, Turkey; 7 IRCCS Ospedale Policlinico San Martino, Genova, Italy; 8 S.S. Trapianto di Cellule Staminali, Torino, Italy; 9 RM Gorbacheva Research Institute, Pavlov University, St. Petersburg, Russian Federation; 10 Institute of Hematology and Blood Transfusion, Prague, Czechia; 11 Turku University Hospital, Turku, Finland; 12 Istituto Clinico Humanitas, Milano, Italy; 13 Department of Hematology and Bone marrow Transplantation GOM Niguarda, Milano, Italy; 14 Anadolu Medical Center Hospital, Kocaeli, Turkey; 15 Imperial College London at Hammersmith Hospital, London, UK; 16 BMT Unit, Hematology Department, Hospital Clínic, Barcelona, Spain; 17 Dipartimento di Scienze di Laboratorio ed Ematologiche-Fondazione Policlinico Universitario Agostino Gemelli-IRCCS, Sezione di Ematologia, Dipartimento di Scienze Radiologiche ed Ematologiche, Università Cattolica del Sacro Cuore, Roma, Italy; 18 Department of Oncology-Hematology, University of Milan and Azienda Socio Sanitaria Territoriale Papa Giovanni XXIII, Bergamo, Italy; 19 Department of Hematology and Cell Therapy, Hospital Saint-Antoine, Sorbonne University, Paris, France; 20 Bone Marrow Transplantation Program, Department of Internal Medicine, American University of Beirut, Beirut, Lebanon; 21 Sorbonne University, Department of Haematology, Saint Antoine Hospital; INSERM UMR 938, Paris, France; 22 Ospedale San Raffaele, Haematology and BMT, Milano, Italy

**Keywords:** Acute myeloid leukaemia, Medical research

## Abstract

Allogeneic hematopoietic stem cell transplantation (HSCT) remains the only curative option for secondary acute myeloid leukemia (sAML). Post-transplant cyclophosphamide has improved graft-versus-host disease (GVHD) prophylaxis, enabling the broader use of alternative donors. For patients lacking a human leukocyte antigen (HLA)-matched donor, haploidentical donor (Haplo) or 9/10 HLA mismatched unrelated donor (MMUD) HSCTs are widely used, yet their relative effectiveness in sAML is uncertain. We retrospectively compared outcomes after Haplo versus MMUD HSCT in adults with sAML in first complete remission transplanted between 2010 and 2022. Among 711 patients, 602 received Haplo and 109 MMUD grafts. Patient and transplant characteristics differed between cohorts, including donor age, conditioning intensity, graft source, and transplant year. Neutrophil recovery was faster after MMUD transplantation, while platelet recovery was comparable. Rates of acute and chronic GVHD, relapse incidence, non-relapse mortality, overall survival, leukemia-free survival, and GVHD-free/relapse-free survival were similar. Reduced intensity conditioning lowered acute GVHD risk, while peripheral blood grafts increased chronic GVHD. Lower Karnofsky score, older age and adverse-risk cytogenetics were adverse prognostic factors. Haplo and MMUD transplantation demonstrated comparable efficacy and safety with post-transplant cyclophosphamide, supporting both approaches as viable alternatives in the absence of an HLA-matched donor.

## Introduction

Secondary acute myeloid leukemia (sAML) arises from antecedent hematologic disorders, such as myelodysplastic or myeloproliferative disease, or as a consequence of prior cytotoxic therapy [1–6]. It is characterized by poor response to conventional treatment, high relapse rates, and a markedly inferior overall survival (OS) compared with de novo AML. These outcomes reflect both the aggressive disease biology and adverse patient-related factors, including older age, adverse-risk cytogenetics, and impaired tolerance to intensive therapy. Allogeneic hematopoietic stem cell transplantation (HSCT) remains the only curative option for sAML; however, historically, outcomes have been poorer in sAML than in de novo AML [7–9].

Advances in graft-versus-host disease (GVHD) prophylaxis, particularly the use of post-transplant cyclophosphamide (PTCy), have expanded donor availability by enabling the safe use of haploidentical donors (Haplo) and improving results with mismatched unrelated donors (MMUD) [10]. Haplo transplantation with PTCy has demonstrated encouraging rates of disease control and acceptable toxicity, suggesting that it may overcome some of the traditionally poor prognostic features of sAML [11–13]. Nevertheless, despite increasing clinical use of both Haplo and MMUD, it remains unclear whether one donor type provides superior outcomes in this high-risk population, as formal comparative analyses are lacking.

To address this knowledge gap, the present study compares the efficacy and safety of Haplo and MMUD HSCT with PTCy in adults with sAML in first complete remission (CR1).

## Methods

### Study design and patient population

This retrospective multicenter study utilized data from the Acute Leukemia Working Party registry of the European Society for Blood and Marrow Transplantation. The European Society for Blood and Marrow Transplantation includes more than 600 transplant centers across Europe, all of which report consecutive transplants and annual follow-up information to a centralized database. Data entry and maintenance follow standardized procedures, and registry quality is ensured through routine validation, cross-checks with national registries, and periodic on-site audits. Since 1990, all patients have provided informed consent permitting use of their anonymized data for research. The study was approved by the Acute Leukemia Working Party institutional review board and conducted in accordance with the Declaration of Helsinki and Good Clinical Practice guidelines.

Eligible patients were adults ( ≥ 18 years) with sAML who underwent a first T-cell–replete allogeneic HSCT from a Haplo or a 9/10 HLA MMUD between 2012 and 2022. Patients were required to be in CR1 and to have received GVHD prophylaxis with PTCy. Conditioning regimens were classified as myeloablative (MAC) or reduced-intensity (RIC) as per previously established criteria [14], and grafts consisted of either bone marrow or peripheral blood stem cells. Exclusion criteria included prior HSCT, combined graft sources, cord blood transplantation, ex vivo T-cell depletion, or transplantation outside CR1.

Collected data included recipient and donor characteristics, disease features (including European LeukemiaNet 2022 cytogenetic risk), antecedent hematologic disorder, transplant year, conditioning intensity, graft source, and GVHD prophylaxis. Acute and chronic GVHD were graded using established criteria [15, 16]. Contributing centers are listed in the Supplementary Appendix.

### Study endpoints and statistical analysis

Quantitative variables were summarized using the median, interquartile range (IQR), and range, while categorical variables were reported as frequencies and percentages. Study endpoints included OS, leukemia-free survival (LFS), relapse incidence (RI), non-relapse mortality (NRM), engraftment, acute and chronic GVHD, and GVHD-free/relapse-free survival (GRFS). All outcomes were measured from the time of transplantation. Engraftment was defined as achieving an absolute neutrophil count ≥0.5 × 10⁹/L for three consecutive days. OS was defined as death from any cause, and LFS as survival without relapse or progression. NRM included all deaths occurring without prior relapse. GRFS was evaluated using modified criteria, with events defined as grade III–IV acute GVHD, extensive chronic GVHD, relapse, or death [17]. Patient, disease, and transplant characteristics were compared using the Mann–Whitney U test for continuous variables and the χ² or Fisher’s exact test for categorical variables. OS, LFS, and GRFS were estimated by the Kaplan–Meier method. Cumulative incidence functions in a competing risks framework were used to calculate RI and NRM, with death in remission treated as a competing event for relapse, and death as a competing event for engraftment. For estimating the cumulative incidence of acute or chronic GVHD, relapse and death were considered competing events. Univariate analyses were performed using the log-rank test for OS, LFS and GRFS, and Gray’s test for cumulative incidence. Multivariate analyses were conducted using the Cox proportional-hazards regression model [18]. All variables differing significantly between the groups, and potential risk factors, were included in the model. To account for inter-center heterogeneity in the effects of characteristics or treatments, a random effect (frailty) term was included in the Cox model [19]. All potential interactions between core variables and other significant variables were evaluated. Univariate and multivariate analyses were conducted on complete case. Results were expressed as the hazard ratio (HR) with a 95% confidence intervals (CI). All *p*-values were two-sided, with a type I error rate of 0.05. Statistical analyses were performed using SPSS version 25.0 (SPSS Inc., Chicago, IL) and R version 4.3.2 (R Core Team).

## Results

### Patient and transplant characteristics

Patient and transplant characteristics are summarized in Table 1. A total of 711 adult patients with sAML in CR1 were included, of whom 602 underwent Haplo HSCT and 109 received an MMUD graft. The distribution of transplant years differed between cohorts (*p* = 0.012). Median age at transplantation was similar (60.6 vs. 60.3 years, *p* = 0.77), as were female donor to male patient, Karnofsky performance status (KPS) score, cytogenetic risk, and patient cytomegalovirus serostatus. The majority of patients had antecedent myelodysplastic or myeloproliferative disease. Median time from diagnosis to HSCT did not differ between groups (5.4 vs. 5.5 months, *p* = 0.70).

**Table 1:** Patient and transplant characteristics.

Variable	Overall N = 711	Haplo N = 602	MMUD N = 109	*p*-value^1^
**Year of transplantation, range**	2019 (2012, 2022)	2019 (2012, 2022)	2019 (2014, 2022)	0.012
**Median FU, y (IQR)**	2.6 (2.3 - 2.9)	2.8 (2.4 - 3)	2 (1.5 - 2.5)	
**Age of the Patient at HCT, y (IQR)**	60.6 (51.2, 66.8)	60.6 (51.6, 66.8)	60.3 (49.9, 66.8)	0.77
**Age of the Donor at HCT, y (IQR)**	35.3 (27.8, 44.3)	36.6 (28.7, 45.3)	30.0 (25.1, 36.8)	<0.001
Missing	17	13	4	
**ELN 2022 cytogenetic risk**				0.49
Favorable	9 (1.6%)	9 (1.9%)	0 (0.0%)	
Intermediate	373 (66.3%)	314 (65.6%)	59 (70.2%)	
Adverse	181 (32.1%)	156 (32.6%)	25 (29.8%)	
Missing	148	123	25	
**Previous diagnosis**				
MDS or MPN	284 (83.5%)	240 (81.8%)	44 (93.6%)	
Other hematological malignancies	17 (5.1%)	16 (5.4%)	1 (2.1%)	
Solid tumor	33 (9.7%)	32 (10.9%)	1 (2.1%)	
Non malignant disease	4 (1.2%)	3 (0.9%)	1 (2.1%)	
Unkown	373 (52.5%)	311 (51.7%)	62 (56.9%)	
**Karnofsky score**				0.92
>= 90	501 (72.9%)	424 (72.9%)	77 (73.3%)	
< 90	186 (27.1%)	158 (27.1%)	28 (26.7%)	
Missing	24	20	4	
**Female donor to male patient**				0.42
No	573 (80.7%)	482 (80.2%)	91 (83.5%)	
Yes	137 (19.3%)	119 (19.8%)	18 (16.5%)	
Missing	1	1		
**CMV antibodies donor to patient**				0.11
Neg to Neg	110 (15.9%)	92 (15.8%)	18 (16.8%)	
Neg to Pos	180 (26.0%)	143 (24.5%)	37 (34.6%)	
Pos to Neg	42 (6.1%)	35 (6.0%)	7 (6.5%)	
Pos to Pos	359 (52.0%)	314 (53.8%)	45 (42.1%)	
Missing	20	18	2	
**RIC or MAC regimen**				0.008
RIC	348 (48.9%)	282 (46.8%)	66 (60.6%)	
MAC	363 (51.1%)	320 (53.2%)	43 (39.4%)	
**Cell source**				<0.001
PB	569 (80.0%)	464 (77.1%)	105 (96.3%)	
BM	142 (20.0%)	138 (22.9%)	4 (3.7%)	
**Median months between diagnosis and HCT (IQR)**	5.4 (3.9, 7.4)	5.4 (3.8, 7.4)	5.5 (4.2, 7.2)	0.70

^1^Pearson’s Chi-squared test; Kruskal-Wallis rank sum test.

*IQR* interquartile range, *FU* follow-up, *y* year, *AML* acute myeloid leukemia, *HSCT* hematopoietic stem cell transplantation, *CMV* cytomegalovirus, *Neg* negative, *Pos* positive, *BM* bone marrow, *PB* peripheral blood, *MAC* myeloablative conditioning, *RIC* reduced intensity conditioning, *MDS* myelodysplastic syndrome, *MPN* myeloproliferative neoplasm, *Haplo* haploidentical donor, *MMUD* mismatched unrelated donor, *ELN* European LeukemiaNet.

Significant differences were observed in transplant characteristics. Donors in the Haplo cohort were significantly older compared to MMUD donors (36.6 vs. 30.0 years, *p* < 0.001), with an overall median donor age of 35.3 years (27.8–44.3). Detailed information on HLA mismatch loci in the MMUD cohort is provided in Table S1. Bone marrow grafts (22.9% vs. 3.7%, *p* < 0.001) and MAC (53.2% vs. 39.4%, *p* = 0.008) were more common in the Haplo group. Thiotepa/busulfan/fludarabine was the most frequently used conditioning in Haplo whereas busulfan/fludarabine predominated in MMUD (Table S2). The most commonly used additional GvHD prophylaxis consisted of a calcineurin inhibitor combined with mycophenolate mofetil in both cohorts (Table S3).

### Engraftment

The cumulative incidence of neutrophil recovery by day 30 was 86.9% after Haplo and 91.8% after MMUD HSCT (Table 2). In multivariate analysis, neutrophil recovery was significantly faster following MMUD transplantation (HR 1.41, 95% CI 1.07–1.85, *p* = 0.016) (Table 3). Platelet recovery ≥20 × 10⁹/L at day 60 was comparable between groups (79.9% vs. 87.5%), with no significant differences on multivariate analysis Tables 2, 3.

**Table 2:** Univariate analysis.

Outcomes % (95% CI)	OS (2 y)	LFS (2 y)	GRFS (2 y)	RI (2 y)	NRM (2 y)
Haplo	58.7 (54.3 - 62.8)	52.4 (48 - 56.6)	41.3 (37 - 45.6)	21.9 (18.4 - 25.6)	25.7 (22.1 - 29.4)
MMUD	64.1 (52.6 - 73.5)	56.9 (45.5 - 66.8)	44.4 (33.4 - 54.8)	26.7 (17.6 - 36.5)	16.4 (9.5 - 24.9)

Outcomes % (95% CI)	aGVHD ≥ II (180 d)	aGVHD ≥ III (180 d)	cGVHD (2 y)	extcGVHD (2 y)
Haplo	27.2 (23.5 - 30.9)	9.8 (7.5 - 12.5)	30.5 (26.5 - 34.5)	10.1 (7.7 - 12.9)
MMUD	28.9 (20.2 - 38.2)	12.4 (6.7 - 19.8)	27.2 (18 - 37.2)	14.1 (7.3 - 23)

Outcomes % (95% CI)	Poly recovery (≥0.5× 10⁹/L) (30 d)	Platelet recovery (≥20× 10⁹/L) (60 d)
Haplo	86.9 (83.9 - 89.4)	79.9 (76.2 - 83.1)
MMUD	91.8 (84 - 95.9)	87.5 (78.8 - 92.8)

*MMUD* mismatched unrelated donor, *Haplo* haploidentical donor, *NRM* non-relapse mortality, *RI* relapse incidence, *LFS* leukemia-free survival, *OS* overall survival, *GVHD* graft-versus-host disease, *GRFS* GVHD-free, relapse-free survival, *y* years, *aGVHD* acute graft-versus-host disease, *cGVHD* chronic graft-versus-host disease, *d* days, *extcGVHD* extensive cGVHD, *CI* confidence interval, *Poly* polymorphonuclear.

**Table 3:** Multivariate analysis.

	OS		LFS		GRFS		RI		NRM	
Variable	HR (95%CI)	*P*-value	HR (95%CI)	*P*-value	HR (95%CI)	*P*-value	HR (95%CI)	*P*-value	HR (95%CI)	*P*-value
**Type of donors: Haplo VS. MMUD**	0.81 (0.52-1.25)	0.343	0.86 (0.59-1.27)	0.457	0.83 (0.58-1.18)	0.288	1.18 (0.7-1.98)	0.531	0.63 (0.34-1.16)	0.141
**Conditioning regimen : RIC VS. MAC**	0.86 (0.63-1.18)	0.36	0.88 (0.66-1.17)	0.387	0.8 (0.62-1.05)	0.108	0.91 (0.59-1.39)	0.655	0.8 (0.53-1.22)	0.309
**Karnofsky score:** **< 90 VS.** **> = 90**	0.69 (0.51-0.92)	0.013	0.73 (0.55-0.96)	0.023	0.75 (0.58-0.97)	0.025	0.9 (0.59-1.38)	0.624	0.62 (0.42-0.91)	0.014
**Age of the Patient at HCT (per 10 years)**	1.16 (1.01-1.33)	0.04	1.09 (0.96-1.23)	0.165	1.12 (1-1.25)	0.062	1.11 (0.92-1.34)	0.261	1.12 (0.94-1.33)	0.215
**Age of the Donor at HCT (per 10 years)**	1.03 (0.91-1.15)	0.664	1 (0.9-1.11)	0.931	1 (0.91-1.1)	0.978	0.89 (0.75-1.05)	0.151	1.07 (0.93-1.23)	0.331
**CMV Donor to patient: Neg to Neg VS. Other**	1.3 (0.87-1.95)	0.191	1.35 (0.93-1.94)	0.111	1.16 (0.85-1.59)	0.359	1.11 (0.67-1.82)	0.684	1.62 (0.93-2.84)	0.09
**Cytogenetic AML classification: Favorable/Int/Missing VS. Adverse**	1.42 (1.06-1.91)	0.019	1.38 (1.05-1.82)	0.02	1.41 (1.1-1.8)	0.007	1.64 (1.11-2.42)	0.014	1.24 (0.84-1.84)	0.279
**Cell source: BM VS. PB**	1.03 (0.71-1.48)	0.891	1.08 (0.77-1.51)	0.653	1.15 (0.84-1.57)	0.372	0.94 (0.58-1.54)	0.82	1.25 (0.77-2.04)	0.372
**Year of transplantation**	0.95 (0.9-1.01)	0.093	0.96 (0.91-1.01)	0.135	0.97 (0.93-1.02)	0.286	0.93 (0.85-1)	0.064	0.98 (0.91-1.06)	0.677

	aGVHD >=II		aGVHD >=III		cGVHD		extcGVHD	
Variable	HR (95%CI)	*P*-value	HR (95%CI)	*P*-value	HR (95%CI)	*P*-value	HR (95%CI)	*P*-value
**Type of donors: Haplo VS. MMUD**	0.88 (0.56-1.38)	0.578	1.3 (0.61-2.76)	0.491	0.54 (0.31-0.95)	0.031	0.91 (0.4-2.1)	0.833
**Conditioning regimen : RIC VS. MAC**	0.59 (0.42-0.84)	0.003	0.87 (0.46-1.62)	0.654	0.98 (0.65-1.49)	0.94	0.43 (0.21-0.86)	0.017
**Karnofsky score:** < **90 VS.** **> = 90**	0.81 (0.58-1.13)	0.218	0.69 (0.39-1.21)	0.194	1.12 (0.74-1.71)	0.589	1.08 (0.56-2.08)	0.825
**Age of the Patient at HCT (per 10 years)**	1.05 (0.9-1.23)	0.533	1.21 (0.91-1.6)	0.194	1.22 (1.04-1.44)	0.016	1.07 (0.81-1.43)	0.624
**Age of the Donor at HCT (per 10 years)**	1.08 (0.94-1.23)	0.279	1.24 (1.01-1.52)	0.044	0.96 (0.83-1.11)	0.572	1 (0.78-1.28)	0.986
**CMV Donor to patient: Neg to Neg VS. Other**	0.98 (0.65-1.47)	0.903	1.22 (0.58-2.55)	0.6	0.85 (0.54-1.35)	0.496	1.05 (0.49-2.23)	0.899
**Cytogenetic AML classification: Favorable/Int/Missing VS. Adverse**	1.08 (0.76-1.53)	0.656	1.4 (0.79-2.49)	0.246	1.57 (1.08-2.28)	0.019	1.54 (0.83-2.87)	0.168
**Cell source: BM VS. PB**	1.48 (0.94-2.35)	0.094	1.59 (0.71-3.57)	0.26	2 (1.19-3.38)	0.009	1.44 (0.59-3.55)	0.423
**Year of transplantation**	1.01 (0.94-1.08)	0.803	1 (0.89-1.12)	0.997	0.94 (0.87-1.02)	0.142	0.97 (0.86-1.1)	0.647

	Poly recovery	
Variable	HR (95%CI)	*P*-value
**Type of donors: Haplo VS. MMUD**	1.41 (1.07-1.85)	0.016
**Conditioning regimen : RIC VS. MAC**	1.07 (0.86-1.34)	0.559
**Karnofsky score:** **< 90 VS.** **> = 90**	0.89 (0.71-1.11)	0.289
**Age of the Patient at HCT (per 10 years)**	0.94 (0.86-1.04)	0.226
**Age of the Donor at HCT (per 10 years)**	1.02 (0.95-1.1)	0.597
**CMV Donor to patient: Neg to Neg VS. Other**	0.91 (0.71-1.17)	0.471
**Cytogenetic AML classification: Favorable/Int/Missing VS. Adverse**	0.97 (0.78-1.19)	0.743
**Cell source: BM VS. PB**	1.15 (0.87-1.51)	0.325
**Year of transplantation**	1 (0.96-1.04)	0.859

*MMUD* mismatched unrelated donor, *Haplo* haploidentical donor, *HSCT* hematopoietic stem cell transplantation, *NRM* non-relapse mortality, *LFS* leukemia-free survival, *OS* overall survival, *GVHD* graft-versus-host disease, GRFS-GVHD-free, relapse-free survival, *HR* hazard ratio, *CI* confidence interval, *aGVHD* acute graftversus-host disease, *AML* acute myeloid leukemia, *CMV* cytomegalovirus, *Int* intermediate, *BM* bone marrow, *PB* peripheral blood, Poly-polymorphonuclear, *Neg* negative, *RIC* reduced intensity conditioning, *MAC* myeloablative conditioning.

### Graft-versus-host disease

**Fig. 1:**
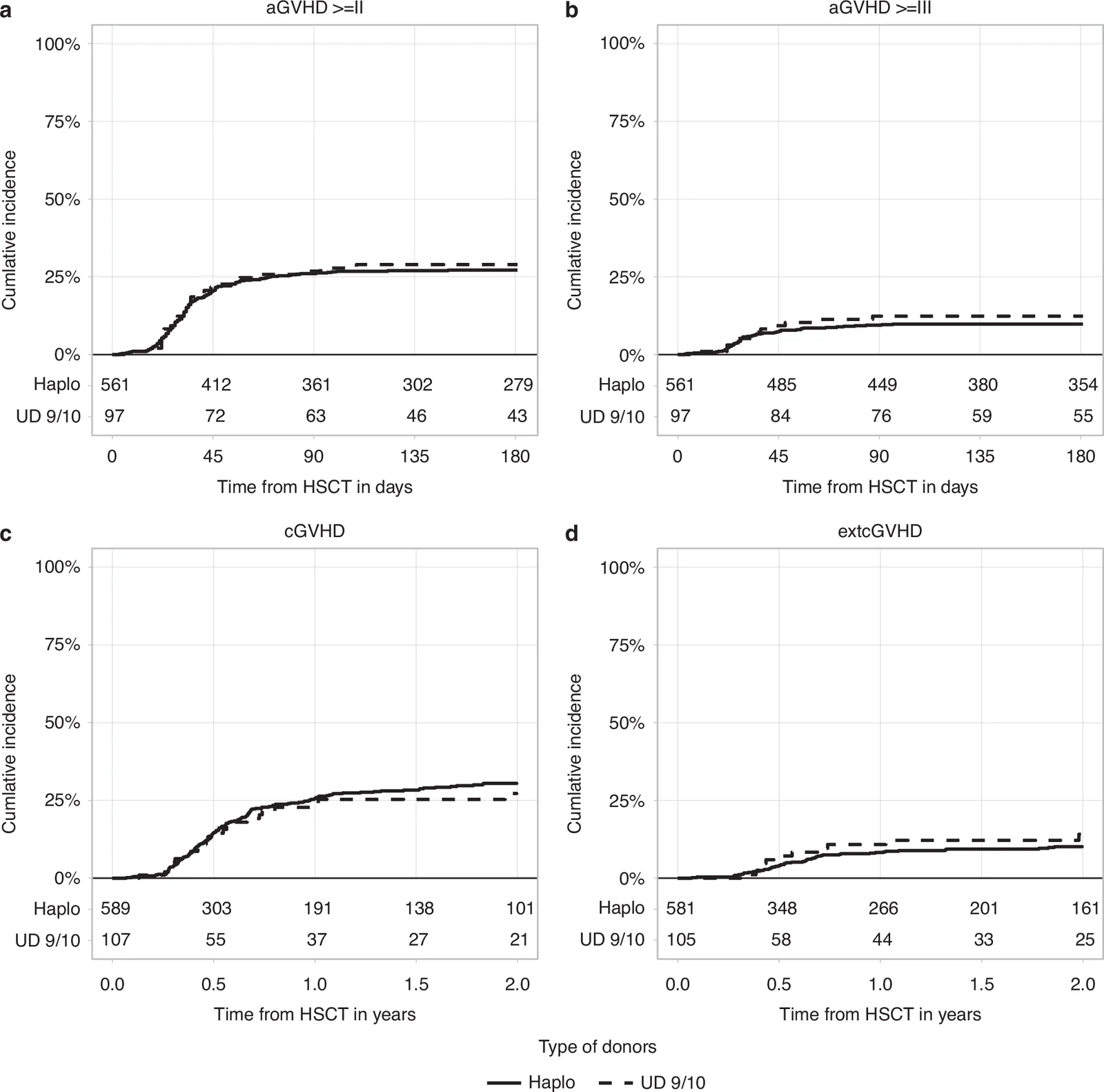
Impact of Donor Type on GVHD in sAML Patients Undergoing Allogeneic HSCT in CR1. Cumulative incidence curves for acute GVHD grade II-IV **a**, acute GVHD grade III-IV **b**, chronic GVHD **c**, and extensive chronic GVHD **d** comparing Haplo and MMUD transplantation. Curves were compared using Gray’s test.

At day 180, the cumulative incidence of grade II–IV acute GVHD was similar between Haplo and MMUD groups (27.2% vs. 28.9%; HR 0.88, *p* = 0.578), as was grade III–IV acute GVHD (9.8% vs. 12.4%; HR 1.30, *p* = 0.491). Two-year cumulative incidence of total chronic GVHD was higher after Haplo transplantation compared with MMUD transplantation (30.5% vs. 27.2%; HR 0.54, 95% CI 0.31–0.95, *p* = 0.031), whereas extensive chronic GVHD did not differ significantly between groups (10.1% vs. 14.1%; HR 0.91, *p* = 0.833) (Fig. 1, Table 2).

MAC was associated with a lower risk of grade II–IV acute GVHD (HR 0.59, *p* = 0.003) and extensive chronic GVHD (HR 0.43, *p* = 0.017). Older patient age was associated with a higher risk of chronic GVHD (HR 1.22, *p* = 0.016), while older donor age independently predicted grade III–IV acute GVHD (HR 1.24, *p* = 0.044). Peripheral blood grafts (HR 2.0, *p* = 0.009) and adverse-risk cytogenetics (HR 1.57, *p* = 0.019) were associated with higher rates of chronic GVHD (Table 3).

### Relapse and non-relapse mortality

**Fig. 2:**
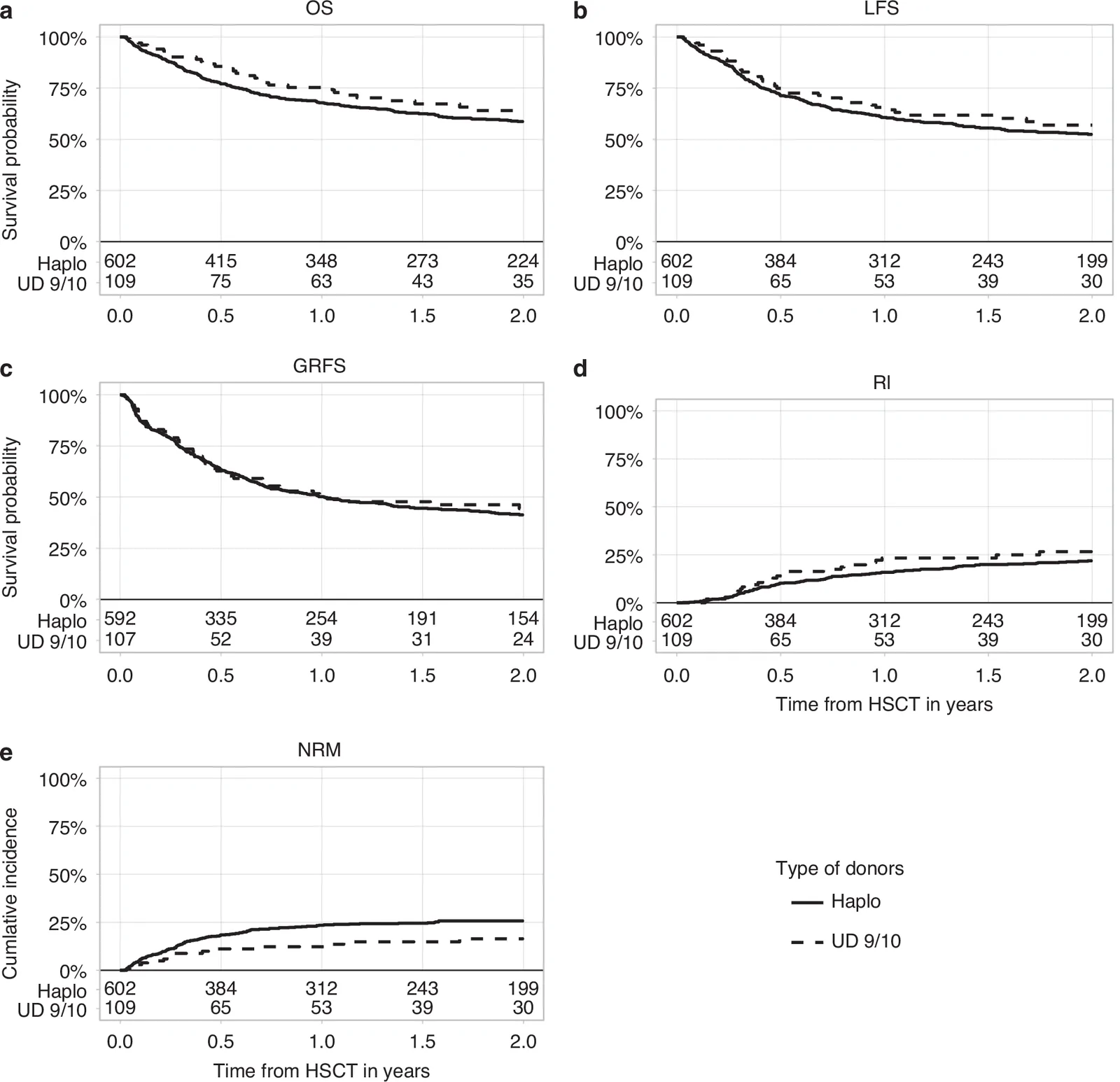
Impact of Donor Type on Outcomes in sAML Patients Undergoing Allogeneic HSCT in CR1. Kaplan-Meier curves for OS **a**, LFS **b**, and GRFS **c** and cumulative incidence curves for RI **d** and NRM **e** comparing Haplo and MMUD transplantation are shown. Survival curves were compared using log-rank tests; Gray’s test assessed cumulative incidence differences.

At two years, RI was comparable between Haplo and MMUD patients (21.9% vs. 26.7%; HR 1.18, *p* = 0.531) (Fig. 2, Table 2). Adverse-risk cytogenetics independently predicted a higher relapse risk (HR 1.64, *p* = 0.014) (Table 3). Two-year NRM was numerically higher after Haplo grafts (25.7% vs. 16.4%) but did not differ significantly (HR 0.63, *p* = 0.141) (Table 2). Lower KPS adversely affected NRM (HR 0.62, *p* = 0.014) (Table 3). Relapse and HSCT-related causes were the leading causes of death in both cohorts (Table S4).

### Survival outcomes

With a median follow-up of 2.6 years, 2-year OS was 58.7% after Haplo and 64.1% after MMUD HSCT (HR 0.81, *p* = 0.343). LFS (52.4% vs. 56.9%; HR 0.86, *p* = 0.457) and GRFS (41.3% vs. 44.4%; HR 0.83, *p* = 0.288) were also comparable between the groups (Fig. 2, Table 2).

In multivariate analysis, adverse-risk cytogenetics was independently associated with inferior OS, LFS, and GRFS. Lower KPS score adversely affected OS, LFS, GRFS, and NRM, while older patient age negatively impacted OS. Donor type was not independently associated with survival outcomes (Table 3).

## Discussion

In this large registry-based analysis of adults with sAML in CR1 undergoing allogeneic HSCT with PTCy-based GVHD prophylaxis, we show that Haplo and MMUD transplantation provide largely comparable outcomes. Aside from faster neutrophil recovery after MMUD transplantation and a lower incidence of chronic GVHD after Haplo transplantation, donor type did not significantly influence relapse risk, NRM, or survival endpoints. Our findings suggest that, in the context of PTCy-based prophylaxis, historical disadvantages associated with donor mismatch may be attenuated [20].

Overall GVHD outcomes were broadly comparable between Haplo and MMUD transplantation, although Haplo transplantation was associated with a higher incidence of chronic GVHD in multivariable analysis. Conditioning intensity and graft source were independently associated with GVHD outcomes, highlighting the importance of transplant strategy optimization in this vulnerable population.

While RI was comparable between Haplo and MMUD transplantation, adverse-risk cytogenetics emerged as the dominant predictor of relapse and inferior survival outcomes [21]. This finding underscores that disease biology remains the primary driver of long-term outcomes in sAML, outweighing the impact of donor selection. Similarly, lower KPS score and older age independently predicted worse survival and higher NRM, highlighting the critical role of patient fitness in transplant decision-making.

Beyond patient age, donor age is increasingly recognized as an independent prognostic factor in allogeneic transplantation [22]. Younger donor age has been associated with improved survival and lower NRM, potentially reflecting a more diverse and functionally competent T-cell repertoire. This consideration is particularly relevant in secondary AML, which predominantly affects older individuals who frequently have age-matched sibling donors. In contrast, both Haplo and MMUD transplantation often allow selection of younger donors. Thus, the availability of the latter in alternative donor platforms may partly offset historical disadvantages associated with HLA mismatch and should be considered when interpreting comparative donor analyses.

Neutrophil recovery was faster after MMUD transplantation, in line with prior reports of delayed neutrophil engraftment after Haploidentical transplantation [23]. The faster neutrophil recovery observed after MMUD transplantation may in part reflect differences in graft source distribution between cohorts, particularly the higher use of bone marrow grafts in the Haplo group. However, this difference did not translate into improved survival or reduced NRM, suggesting limited clinical relevance in the setting of effective supportive care and GVHD prophylaxis.

The strengths of this study include its large sample size, homogenous disease status (CR1), exclusive use of PTCy-based prophylaxis, and comprehensive multivariable modeling accounting for center effects. Nevertheless, limitations inherent to retrospective registry analyses apply, including potential residual confounding, missing molecular data beyond cytogenetics, including TP53 mutational status, and imbalances in conditioning regimens and graft source between groups. The smaller size of the MMUD cohort limits power to detect modest differences. Median follow-up also differed between cohorts, which may have influenced the assessment of later outcomes such as chronic GVHD and relapse, although the difference was less than one year and major outcomes were analyzed at predefined 2-year time points. The MMUD cohort was analyzed as a single group despite likely heterogeneity regarding the specific HLA mismatch locus. Owing to the limited sample size of the MMUD cohort, more detailed subgroup analyses according to mismatch locus were not feasible. We lacked detailed comorbidity data such as the HCT-CI, relying instead on the Karnofsky score, and did not have comprehensive information on pre-transplant chemotherapy, including prior CPX-351 exposure, precluding assessment of its potential impact on post-transplant toxicity. Information allowing reliable distinction between AML arising from antecedent hematologic disorders and therapy-related AML was incomplete for a substantial proportion of patients. MRD data at transplantation and details regarding MRD assessment methods were incomplete, precluding robust analyses of the impact of MRD status on post-transplant outcomes. Detailed and consistent data on post-transplant maintenance therapies were also not available for all patients and therefore could not be incorporated into the present analysis.

In conclusion, our findings indicate that Haplo and MMUD HSCT yield equivalent outcomes in patients with sAML in CR1 when PTCy-based GVHD prophylaxis is used. Given the urgency of transplantation in sAML and frequent lack of matched donors, donor availability and timing rather than donor hierarchy should guide clinical decision-making. Future studies incorporating molecular risk stratification and post-transplant interventions are needed to further improve outcomes in this high-risk population.

## Supplementary information

Supplementary Appendix

## Data Availability

AN, ATF, MM and FC had full access to all the study data (available upon data-specific request).
