# Supplementary material for: T-cell replete haploidentical transplantation compared to mismatched unrelated allogeneic transplantation with post-transplantation cyclophosphamide in patients with secondary acute myeloid leukemia in first complete remission: A study from the ALWP/EBMT
Source: Bone Marrow Transplant. 2026 Jun 27;61(9):1196–203. doi: 10.1038/s41409-026-02951-9 (PMC13569454; doi:10.1038/s41409-026-02951-9)
Supplement: Supplementary file 1 [file 41409_2026_2951_MOESM1_ESM.docx]

**Supplementary Appendix**

**Contributing Centers**

Programme de Transplantation&Therapie Cellulaire, Marseille, France; IRCCS San Raffaele Hospital Scientific Institute, Milano, Italy; Medicana International Hospital Istanbul, Istanbul, Turkey; IRCCS Ospedale Policlinico San Martino, Genova, Italy; S.S.C.V.D Trapianto di Cellule Staminali, Torino, Italy; RM Gorbacheva Research Institute, Pavlov University, St. Petersburg, Russia; Institute of Hematology and Blood Transfusion, Prague, Czechia; Turku University Hospital, Turku, Finland; Istituto Clinico Humanitas, Milano, Italy; ASST GRANDE OSPEDALE METROPOLITANO NIGUARDA, Milano, Italy; Anadolu Medical Center Hospital, Kocaeli, Turkey; Imperial College Hammersmith London, London, United Kingdom; Hospital Clinic, Barcelona, Spain; Universita Cattolica S. Cuore, Rome, Italy; ASST Papa Giovanni XXIII, Bergamo, Italy; Departement d'Oncologie, Service d'Hematologie, Geneva, Switzerland; CHU Bordeaux, Hopital Haut-Leveque, Pessac, France; Hospital U. Marques de Valdecilla, Santander, Spain; University Hospital | Basel, Basel, Switzerland; Oslo University Hospital, Rikshospitalet, Oslo, Norway; CHU Grenoble Alpes - Universite Grenoble Alpes, Grenoble, France; CHU Nice - Hopital de l'ARCHET I, Nice, France; Hospital Ramon y Cajal, Madrid, Spain; Centre Hospitalier Lyon Sud, Pierre Bénite cedex, France; Hospital Clinico, Salamanca, Spain; Universitaetsklinikum Dresden, Dresden, Germany; Hospital Gregorio Maranon, Madrid, Spain; University Hospital Frankfurt - Goethe University, Frankfurt Main, Germany; ALBERTS CELLULAR THERAPY, Pretoria, South Africa; University Hospital La Fe, Valencia, Spain; Azienda Ospedali Riuniti di Ancona, Ancona, Italy; Demiroglu Bilim University Florence Nightingale Hospital, Istanbul, Turkey; Klinikum Augsburg, Augsburg, Germany; Saint-Louis Hospital, BMT Unit, Paris, France; Medizinische Universitaet Wien, Vienna, Austria; CHU de Lille, Lille, France; Hospital Clinico de Valencia, Valencia, Spain; European Institute of Oncology, Milano, Italy; University Hospital Eppendorf, Hamburg, Germany; Hospital Santa Creu i Sant Pau, Barcelona, Spain; Universite Paris IV, Hopital la Pitie-Salpetriere, Paris, France; Klinikum Grosshadern, München, Germany; AORMN Hospital, Pesaro, Italy; University Medical Center Groningen (UMCG), Groningen, Netherlands; Azienda Ospedaliera Universitaria Integrata Verona, Verona, Italy; Arcispedale S. Maria Nuova, Reggio Emilia, Italy; Azienda Sanitaria Universitaria Friuli Centrale, Udine, Italy; Azienda Ospedaliero Universitaria Pisana, Pisa, Italy; University Clinical Centre in Gdansk, Gdansk, Poland; San Matteo Pavia Transplant Programme, Pavia, Italy; USD Trapianti di Midollo, Adulti, Brescia, Italy; RVI Newcastle, Newcastle, United Kingdom; Hannover Medical School, Hannover, Germany; Hospital San Maurizio, Bolzano, Italy; University Hospital Center Rebro, Zagreb, Croatia; University Hospitals Bristol and Weston NHSFT, Bristol, United Kingdom; TUM Universitatsklinikum, Klinikum rechts der Isar, München, Germany; University of Napoli, Napoli, Italy; University of Saarland, Homburg, Germany; University Hospital Erlangen, Erlangen, Germany; Centro Trapianti Unico Di CSE Adulti e Pediatrico A. O Brotzu, Cagliari, Italy; Unita Operativa di Ematologia e Trapianto di cellule staminali, Lecce, Italy; Centre Henri Becquerel, Rouen, France; CHRU Limoges, Limoges, France; Antwerp University Hospital (UZA), Antwerp, Belgium; Universitaetsmedizin Mannheim, Mannheim, Germany; Hopital Necker Adults, Paris, France; Hospital Sirio-Libanes, São Paulo, Brazil; Institut Jules Bordet, Brussels, Belgium; Institut de Cancerologie Lucien Neuwirth, Saint Etienne, France; Hopital Henri Mondor, Creteil, France; Hopital La Miletrie, Poitiers, France; University Hospital Innsbruck, Innsbruck, Austria; CHU ESTAING, Clermont, France; Ospedale S. Camillo-Forlanini, Rome, Italy; Ospedale Infantile Regina Margherita, Torino, Italy; Fundeni Clinical Institute Adults, Bucharest, Romania; University of Cologne, Cologne, Germany; Ospedale San Gerardo, Monza, Italy; Grande Ospedale Metropolitano Bianchi Melacrino Morelli, Reggio Calabria, Italy; Elisabethinen Hospital, Linz, Austria; Az. Ospedaliera S. Croce e Carle, Cuneo, Italy; ICO-Hospital Universitari Germans Trias i Pujol, Badalona, Spain; Evangelismos Hospital, Athens, Greece; AZ Delta, Roeselare, Belgium; ICANS - Institut de cancerologie Strasbourg Europe, Strasbourg, France; Umea University Hospital, Umeå, Sweden; Kings College Hospital London, London, United Kingdom; A.O.R.N. 'SAN.G MOSCATI', Avellino, Italy; Gazi University Faculty of Medicine, Ankara, Turkey; Klinikum Frankfurt (Oder) GmbH, Frankfurt Oder, Germany; Karolinska University Hospital, Stockholm, Sweden; Royal Marsden Hospital, Leeds, United Kingdom; University College London Hospital, London, United Kingdom; Nijmegen Medical Centre, Nijmegen, Netherlands; Erasmus MC Cancer Institute, Rotterdam, Netherlands; Ospedale Civile, Pescara, Italy; CHU CAEN, Caen, France; University Hospital | Essen, Essen, Germany; Fondazione IRCCS - Ca Granda, Milano, Italy; Klinikum Karlsruhe gGmbH, Karlsruhe, Germany; Azienda Ospedaliera Universitaria Careggi, Firenze, Italy; U.O.S.A Centro Trapianti e Terapia Cellulare, Siena, Italy; Martin-Luther-Universitaet Halle-Wittenberg, Halle (Saale), Germany; HUCH Comprehensive Cancer Center, Helsinki, Finland; IRCCS, Casa Sollievo della Sofferenza, San Giovanni, Italy; Hospital de Gran Canaria Dr Negrin, Las Palmas, Spain; George Papanicolaou General Hospital, Thessaloniki, Greece; University Hospital Maastricht, Maastricht, Netherlands; King Hussein Cancer Centre Adult BMT Program, Amman, Jordan; VU University Medical Center, Amsterdam, Netherlands; CHU - Institut Universitaire du Cancer Toulouse, Toulouse, France; Universitair Ziekenhuis Brussel, Brussels, Belgium; C.H.R.U de Brest, Brest, France; Gustave Roussy Cancer Campus, Villejuif, France; CHRU NANCY, Vandoeuvre les Nancy, France; Clinica Puerta de Hierro, Madrid, Spain; Hospital Universitario La Paz, Madrid, Spain; Hospital Morales Meseguer, Murcia, Spain; Institut Catala d'Oncologia - Hospital Duran i Reynals, Barcelona, Spain; Hospital Universitario Virgen del Rocio, Sevilla, Spain; H SS. Antonio e Biagio, Alessandria, Italy; Ospedale San Carlo, Potenza, Italy; Perrino Hospital, Brindisi, Italy; Medical School University of Salerno, Salerno, Italy; Mazzoni Hospital, Ascoli Piceno, Italy; Robert_Bosch_Krankenhaus, Stuttgart, Germany; University Hospital Ostrava, Ostrava, Czechia; Tel Aviv Sourasky Medical Center, Tel Aviv, Israel; Hospital Guglielmo da Saliceto, Piacenza, Italy; Leiden University Hospital, Leiden, Netherlands; University Hospital | Zurich, Zürich, Switzerland; Hopital Jean Minjoz, Besancon, France; Hospital de la Princesa, Madrid, Spain; IRCCS Azienda Ospedaliero-Universitaria di Bologna, Bologna, Italy; Glasgow Royal Infirmary, Glasgow, United Kingdom; CHU Nantes, Nantes, France; Hopital Bretonneau, Tours, France; Birmingham Centre for Cellular Therapy and Transplant (BCCTT), Birmingham, United Kingdom; Inst. Portugues Oncologia, Lisboa, Portugal; LKH - University Hospital Graz, Graz, Austria; Fundacion Jimenez Diaz, Madrid, Spain; ZNA, Antwerpen, Belgium; Birmingham Centre for Cellular Therapy and Transplant (BCCTT), Birmingham, United Kingdom; U.O.D Trapianti di midollo osseo, Palermo, Italy; Hospital Alvaro Cunqueiro, Vigo, Spain; King Fahad Specialist Hospital, Dammam, Saudi Arabia; King Abdul - Aziz Medical City, Riyadh, Saudi Arabia; Azienda Ospedaliero Universitaria di Modena Policlinico, Modena, Italy; Del-pesti Centrumkorhaz, Budapest, Hungary; Hospital Univ. Virgen de las Nieves, Granada, Spain; Addenbrookes Hospital Cambridge, Cambridge, United Kingdom; Hospital Universitario de Navarra, Pamplona, Spain; Hospital Vall d'Hebron, Barcelona, Spain; Cardarelli Hospital, Napoli, Italy; Ankara University Faculty of Medicine, Ankara, Turkey; Hospital Universitario Central de Asturias, Oviedo, Spain; University of Debrecen Clinical Center, Debrecen, Hungary; CHRU, Angers, France; Centre Hospitalier Universitaire de Rennes, Rennes, France; Centre Pierre et Marie Curie, Algiers, Algeria; Charles University Hospital, Plzeň, Czechia; University of Liege, Liege, Belgium; University Hospital | Linkoeping, Linkoeping, Sweden; Ghent University Hospital, Gent, Belgium; Tartu University Hospital, Tartu, Estonia; University Medical Center Mainz, Mainz, Germany; University Regensburg, Regensburg, Germany; Sezione di Ematologia, Perugia, Italy; S. Bortolo Hospital, Vicenza, Italy; University of Freiburg, Freiburg, Germany.

**Supplemental Table S1. HLA mismatch loci in the MMUD cohort**

| **HLA mismatch locus** | **n (%)** |
| --- | --- |
| HLA-A | 46 (42.2%) |
| HLA-B | 20 (18.3%) |
| HLA-C | 18 (16.5%) |
| HLA-DR | 11 (10.1%) |
| HLA-DQ | 14 (12.8%) |

Abbreviations: MMUD- mismatched unrelated donor; HLA- Human Leukocyte Antigen

**Supplemental Table S2. Conditioning regimens**

| **Variable** | **Overall** | **Haplo** | **MMUD** |
| --- | --- | --- | --- |
|  | N = 711 | N = 602 | N = 109 |
| TBI-based | 119 (16.7%) | 103 (17.1%) | 16 (14.7%) |
| TBF-based | 301 (42.3%) | 280 (46.5%) | 21 (19.3%) |
| TreoFlu-based | 71 (10.0%) | 56 (9.3%) | 15 (13.8%) |
| BuFlu-based | 147 (20.7%) | 113 (18.8%) | 34 (31.2%) |
| BuCy-based | 12 (1.7%) | 5 (0.8%) | 7 (6.4%) |
| FluMel-based | 50 (7.0%) | 37 (6.1%) | 13 (11.9%) |
| Other | 11 (1.5%) | 8 (1.3%) | 3 (2.8%) |

Abbreviations: MMUD- mismatched unrelated donor; Haplo-haploidentical donor; TBI-total body irradiation; Mel-melphalan; Bu-busulfan; Flu-fludarabine; Cy-cytoxan; TBF-thiotepa, Bu and Flu; Treo-treosulfan

**Supplemental Table S3. Associated GVHD prevention**

| **Variable** | **Overall** | **Haplo** | **MMUD** |
| --- | --- | --- | --- |
|  | N = 711 | N = 602 | N = 109 |
| CSA-based | 15 (2.1%) | 6 (1.0%) | 9 (8.3%) |
| MMF-based | 17 (2.4%) | 14 (2.3%) | 3 (2.8%) |
| MMF+CSA-based | 393 (55.3%) | 336 (55.8%) | 57 (52.3%) |
| MMF+CSA+TACRO-based | 12 (1.7%) | 10 (1.7%) | 2 (1.8%) |
| MMF+SIRO-based | 34 (4.8%) | 29 (4.8%) | 5 (4.6%) |
| MMF+TACRO-based | 193 (27.1%) | 169 (28.1%) | 24 (22.0%) |
| MTX+CSA-based | 9 (1.3%) | 8 (1.3%) | 1 (0.9%) |
| MTX+TACRO-based | 1 (0.1%) | 1 (0.2%) | 0 (0.0%) |
| TACRO-based | 22 (3.1%) | 14 (2.3%) | 8 (7.3%) |
| Other | 15 (2.1%) | 15 (2.5%) | 0 (0.0%) |

Frequency (%)

Abbreviations: GVHD-graft versus host disease; MMUD- mismatched unrelated donor; Haplo-haploidentical donor; CSA- cyclosporine A; MMF- mycophenolate mofetil; SIRO- sirolimus; TACRO- tacrolimus; MTX-methotrexate

**Supplemental Table S4. Cause of death**

| **Main cause of death** | **Haplo** N = 252 | **MMUD** N = 35 |
| --- | --- | --- |
| Relapse | 100 (39.7%) | 18 (51.4%) |
| HSCT related | 134 (53,1%) | 15 (42.9%) |
| Secondary malignancy | 2 (0.8%) | 1 (2.9%) |
| Other | 16 (6.3%) | 1 (2.9%) |

Abbreviations: HSCT-hematopoietic stem cell transplantation; MMUD-mismatched unrelated donor; Haplo-haploidentical donor
